# Application of epicutaneo-cava catheters with 24G indwelling needles in very low birth weight infants: a safe and simple innovative technique

**DOI:** 10.3389/fped.2023.1172164

**Published:** 2023-05-25

**Authors:** Qin Wang, Feixiang Luo, Xiaoyan Fan, Xiaoying Cheng, Xiaolu Ma, Liping Shi, Pei Zhang

**Affiliations:** ^1^ NICU, Children’s Hospital, Zhejiang University School of Medicine, Hangzhou, China; ^2^ Quality Management Office, Children’s Hospital, Zhejiang University School of Medicine, Hangzhou, China

**Keywords:** ECC, VLBW, 24G indwelling needle, conventional insertion, catheter

## Abstract

**Background:**

Epicutaneo-cava catheter (ECC) is an ideal venous access for very low birth weight (VLBW) infants. However, because veins of VLBW infants are thin, ECC catheter is difficult to insert, and the success rate of puncture is low. This study aimed to use ECC with 24G indwelling needles to improve the outcomes of VLBW infants.

**Methods:**

This retrospective study enrolled 121 VLBW infants (birthweight <1,500 g) who required ECC catheterization and were admitted to the Neonatal Intensive Care Unit of the Children's Hospital of Zhejiang University School of Medicine between January 2021 and December 2021. The patients were divided into the indwelling needle group and the conventional technique group according to the technique of ECC. The demographic and treatment data of the two groups were collected, and the success rate of first attempt cannulation of ECC and catheter-related complications of the two groups were analyzed and compared.

**Results:**

There were no significant differences in gender, age, and body weight between the two groups on the day of ECC insertion and venipuncture site. It can be seen through model analysis that the success rate of first-attempt cannulation of ECC in the indwelling needle group was significantly higher than in the conventional technique group. In contrast, average catheterization time and catheterization-related bleeding risk in the indwelling needle group were significantly lower than in the conventional technique group (*p* = 0.00,and 0.00, respectively). Infection during catheter placement, indwelling catheter duration and catheter-related infection between the two groups (*p* > 0.05).

**Conclusion:**

Application of ECC with 24G indwelling needles in VLBW infants can improve the success rate of first attempt cannulation of ECC, reduce the time of catheterization and the risk of bleeding, which may be popularized for widespread application.

## Introduction

Very low birth weight (VLBW) refers to premature infants with a birth weight < 1,500 g, and extremely low birth weight (ELBW) refers to premature infants with a birth weight < 1,000 g. These premature infants need long-term parenteral nutrition (PN) due to incomplete gastrointestinal development and function. Therefore, establishing an appropriate venous access plays an important practical role in the treatment of VLBW infants. Epicutaneo-cava catheter (ECC) has the characteristics of safety, reliability, hypertonicity,and long retention time,which not only reduces excessive stimulation but also ensures the supply of intravenous nutrition, providing ideal venous access in VLBW infants (1–3).

The conventional method of ECC is to use the sheath to puncture into the peripheral blood vessel, withdraw the steel needle, insert the ECC into the peripheral blood vessel through the hollow channel of the sheath, and enter the central vein along with the blood vessel. Thereafter, the sheath is removed and the catheter is secured (4, 5). At present, the smallest diameter of commercially available ECC is 1.9 Fr (French, 1 Fr = 0.33 mm, 1.9 Fr = 0.63 mm)in China, which is also the only model available for VLBW infants. The sheath of this type of catheter is 20G, and the inner diameter is 1.2 mm. However, because the blood vessels of VLBW infants are immature and the structure and shape of peripheral medium veins that can be used for ECC are similar to adult venules with narrow diameter, thin vessel wall, and less elastic fibers, collagen fibers and smooth muscles, the external force of the puncture can easily cause the rupture of the blood vessel wall, which leads to massive bleeding at the puncture site of ECC after the sheath is torn off (6).

NICU experts are actively trying various new methods to solve this problem (7, 8). Our team established a new ECC method in this study. INS found (9, 10) that 24G indwelling needles are more suitable for neonates. Given the thin blood vessels of VLBW infants and the thick sheath, we used a 24G indwelling needle to replace the sheath for ECC and inserted the catheter using the stylet in the puncture package as a structural support to reduce the problems associated with ECC, such as destruction of the peripheral medium veins, bleeding, and multiple punctures.

## Ethics

The study protocol was approved by the Institutional Review Board of the Children's Hospital of Zhejiang University School of Medicine. Informed consent was waived due to the retrospective study design. All procedures in this study followed the relevant guidelines and regulations.

## Patient data and methods

This retrospective study was conducted in the NICU of the Children's Hospital affiliated to Zhejiang University School of Medicine between January 2021 and December 2021.This study retrospectively analyzed the clinical data of 121 VLBW infants who were admitted to our department between January 2021 and December 2021 and underwent ECC, and compared the catheterization effect between the indwelling needle group and the conventional technique group.

Inclusion criteria were: VLBW infants who underwent ECC within seven days of birth; birth weight < 1,500 g; 1.9 Fr ECC catheter was used (Medcom, USA). Exclusion criteria were: Patients with a history of ECC in other hospitals; patients with congenital heart disease, omphalocele, peritonitis and other diseases; patients with infection indications during puncture. Catheterization was completed by senior specialist nurses (Wang Qin and Luo Feixiang) with many years of NICU ECC experience. Before ECC, the nurse explained the pros and cons to the family members of the children, and then the family members decided on the technique of ECC. The patients were divided into the indwelling needle group and the conventional technique group according to the chosen technique of ECC.

### Insertion technique of the indwelling needle group

**Figure 1:**
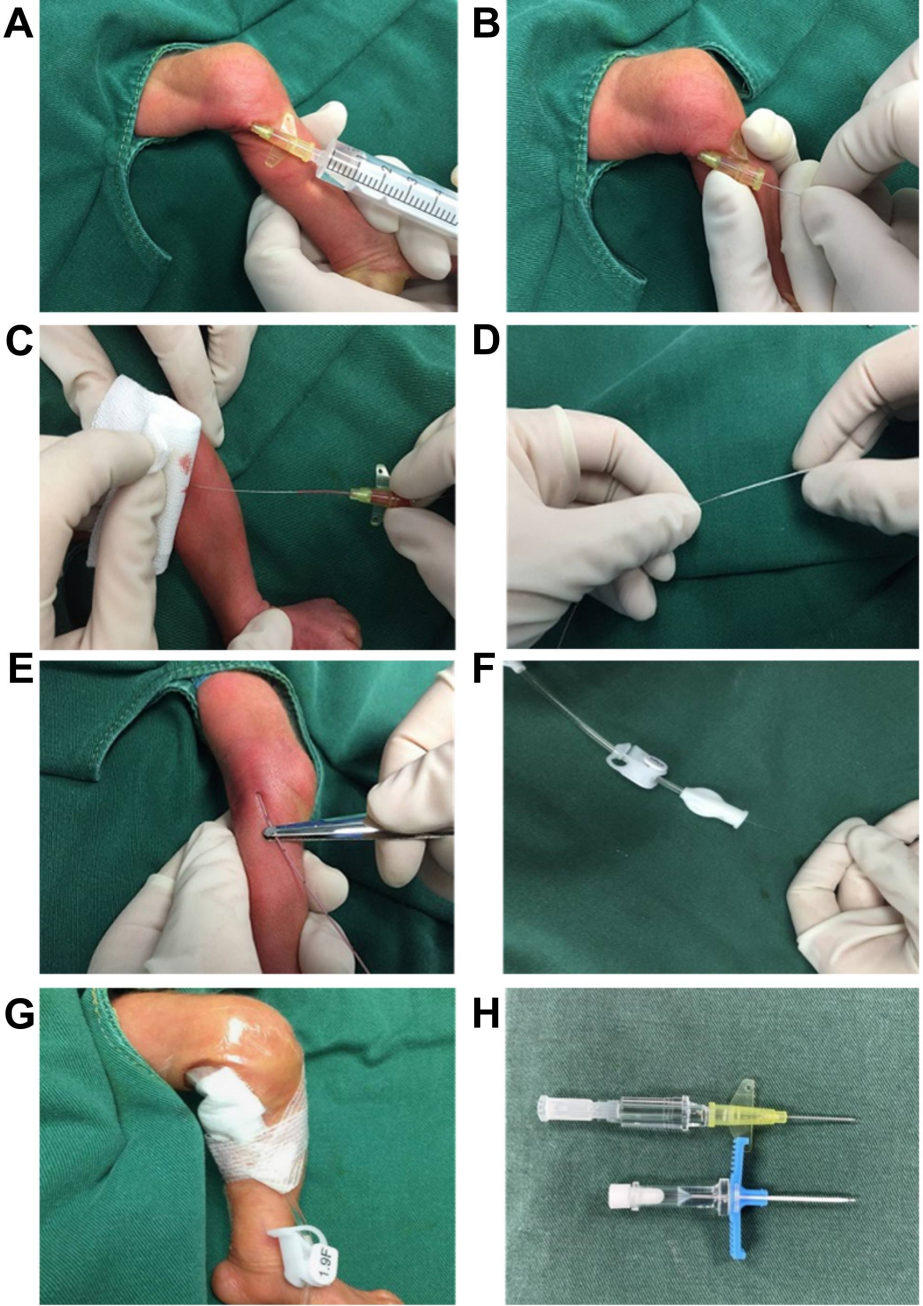
The insertion of the 1.9Fr PICC using a 24G indwelling needle (**A–H**).

A 24G indwelling needle was used for puncture, and the catheter was inserted into the body through the stylet of 1.9 Fr ECC. The specific steps were as follows: ① The puncture site was sterilized, and a 24G straight (with wing) indwelling needle (Bidi, Singapore) was inserted into the selected vein (Figure 1A). ② The ECC's own stylet was inserted into the vein through the indwelling needle, and the stylet was pushed into the vein about 0.5–1 cm beyond the length of the indwelling needle (Figure 1B). ③ The indwelling needle was withdrawn (Figure 1C). ④ The ECC catheter entered the blood vessel with the support of the stylet (Figure 1D,E). ⑤ The stylet was withdrawn into the catheter, the catheter was pushed to the predetermined length, and the stylet was retracted (Figure 1F). ⑥ The catheter was fixed with a sterile applicator (Figure 1G).

### Insertion technique of the conventional technique group

The conventional technique group adopted the classical puncture method such that the sheath in the ECC catheter kit was used to puncture for inserting the ECC. After ECC, bedside B-ultrasound or x-ray was used to locate and confirm the position of the tip. Maintenance of ECC was performed according to the standard ECC maintenance procedure.

The data on gender, age (in days) on the day of ECC, body weight on the day of ECC, diagnosis, venipuncture site, CRP and PLT values during catheter placement, times of punctures, catheter puncture time of ECC, and catheter indwelling time was collected from the two groups. Catheter-related complications, including bleeding risk after catheterization, phlebitis, and catheter-related infection, were recorded. Outcomes of CRBSI and CLABSI were considered as indicators of catheter-related infection.

The success rate of the first attempt cannulation means that the catheter is inserted to the required length after the puncture, indicating successful cannulation. Puncture failure means that the puncture needle does not touch the corresponding vein and exits the puncture point during the puncture. The catheter puncture time of ECC refers to the time from the start of the disinfection of the drape to the proper fixation of the catheter, which is calculated in minutes. The bleeding risk after catheterization refers to the application of 1 cm*1 cm sterile gauze to stop bleeding after ECC. According to INS, the sterile dressing needs to be replaced within 48 h, and the times of replacements within 48 h after tip positioning is used as a statistical indicator. Catheter-related bloodstream infection (CRBSI) is defined according to clinical practice guidelines (11) such that the same organism grows from at least one percutaneous blood culture and from a culture of the catheter tip or that two blood samples are drawn (one from a catheter hub and the other from a peripheral vein). Central line-associated bloodstream infection (CLABSI) is defined as bacteremia in a patient who had at least one positive blood culture result obtained from a peripheral vein, clinical manifestations of infection and no other apparent source of bloodstream infection except catheter placement in the 48-hour period before blood stream infection, according to the Centers for Disease Control and Prevention (CDC) (12, 13).

### Statistical analysis

Statistical analysis of data was performed using SPSS 23.0 software (IBM SPSS Statistics, Armonk, NY). The measurement data was analyzed by Kolmogorov-Smirnov test and expressed as mean ± standard deviation (xˉ ± s), and the comparison between the two groups was performed by Student's t-test. The categorical data was expressed as the number of cases or percentage (%), and the comparison between the two groups was performed by chi-square test. A *p*-value < 0.05 was considered statistically significant.

Multivariate logistic regression model was used to analyze the association between the method of ECC and the success rate of first attempt cannulation of ECC. Multivariate linear regression model was used to analyze the association between the method of PICC and Catheter indwelling duration. In these two models, gender, age on the day of ECC, and body weight on the day of ECC were adjusted.

## Results

A total of 121 children were enrolled in this study. There were 61 children in the indwelling needle group, with 29 males (47.5%) and 32 females (52.5%). The average age on the day of ECC was 4.38 ± 1.99 (1, 7) days, and the average body weight on the day of ECC was 0.98 ± 0.24 (0.56, 1.42) kg. There were 60 children in the conventional technique group, with 31 males (51.7%) and 29 females (48.3%). The average age on the day of ECC was 4.58 ± 1.80 (1, 7) days, and the average body weight on the day of ECC was 1.03 ± 0.23 (0.58, 1.49) kg. The two groups were diagnosed as premature infants, with VLBW and neonatal respiratory distress syndrome. The puncture sites of ECC in the two groups mainly included the basilic vein, saphenous vein, axillary vein, popliteal vein and temporal vein. There were no significant differences between the two groups in gender, age (in days) on the day of ECC, body weight on the day of ECC, venipuncture site, infection during catheter placement and catheter indwelling duration (*p* > 0.05) (Table 1).

**Table 1:** Comparison of general data, infection during catheter placement, catheter indwelling duration and catheter-related infection between the two groups.

Items	The indwelling needle group (*n* = 61)	The conventional technique group (*n* = 60)	*p-*value
**Gender**			
Male	29 (47.5%)	31 (51.6%)	0.653
Female	32 (52.5%)	29 (48.4%)	
Age (in days) on the day of ECC (day)	4.38 ± 1.99	4.58 ± 1.80	0.550
Body weight on the day of ECC (kg)	0.98 ± 0.24	1.03 ± 0.23	0.193
**Venipuncture site**			
Basilic vein	13 (21.3%)	18 (30.0%)	0.309
Sphenous vein	29 (47.5%)	22 (36.7%)	
Axillary vein	7 (11.5%)	12 (20.0%)	
Popliteal vein	4 (6.6%)	2 (3.3%)	
Temporal vein	8 (13.1%)	5 (8.3%)	
Puncture failure	0	1 (1.7%)	
**Blood routine examination during ECC**			
CRP(mg/L)	5.41 ± 17.19	2.07 ± 4.45	0.147
PLT (*10^9^/L)	203.69 ± 97.14	205.95 ± 90.55	0.895
Catheter indwelling duration	18.58 ± 5.32	21.64 ± 6.47	0.206

A total of 61 children were enrolled in the indwelling needle group. Success rate of first attempt cannulation of ECC in the indwelling needle group was 91.8% (56/61). There were five cases (8.2%) with ≥2 successful ECC. A total of 60 children were enrolled in the conventional technique group. Success rate of first attempt cannulation of ECC in the conventional technique group was 71.7% (43/60). There were 16 cases (26.6%) with ≥2 successful ECC, and one case (1.7%) with puncture failure. The success rate of the puncture in the indwelling needle group was significantly higher than that of the conventional technique group. Through model analysis, it can be seen that the success rate of first attempt cannulation in the the indwelling needle group is 4.42 times that of the conventional technique group, which is significantly improved, while the puncture time is significantly decreased.At the same time, due to the influence of gender, age and weight of the baby, we adjusted the model,the success rate of first attempt cannulation in the the indwelling needle group is 4.91 times that of the conventional technique group.After removing these factors, the results were still not affected. Therefore, the relationship between the two groups were very stable and the results were reliable (Table 2).

**Table 2:** Comparison of puncture times between the two groups.

Items	The indwelling needle group (*n* = 61)	The conventional technique group (*n* = 60)	Crude OR (95%CI) or β (95%CI)^a^	aOR (95%CI) or β (95%CI)^b^
Success rate of first attempt cannulation of ECC, n/N	56/61 (91.8%)	43/60 (71.7%)	4.42 (1.514, 12.953)	4.91 (1.325, 18.271)
Catheter puncture time, min	34.9 ± 22.7	44.9 ± 29.6	−10.032 (−18.047, −2.016)	−8.805 (−16.544, −1.066)

^a^ Reference is the conventional technique group.

^b^ Reference is the conventional technique group. Gender, age on the day of ECC, and body weight on the day of ECC were adjusted. aOR (95%CI) was presented.

**Figure 2:**
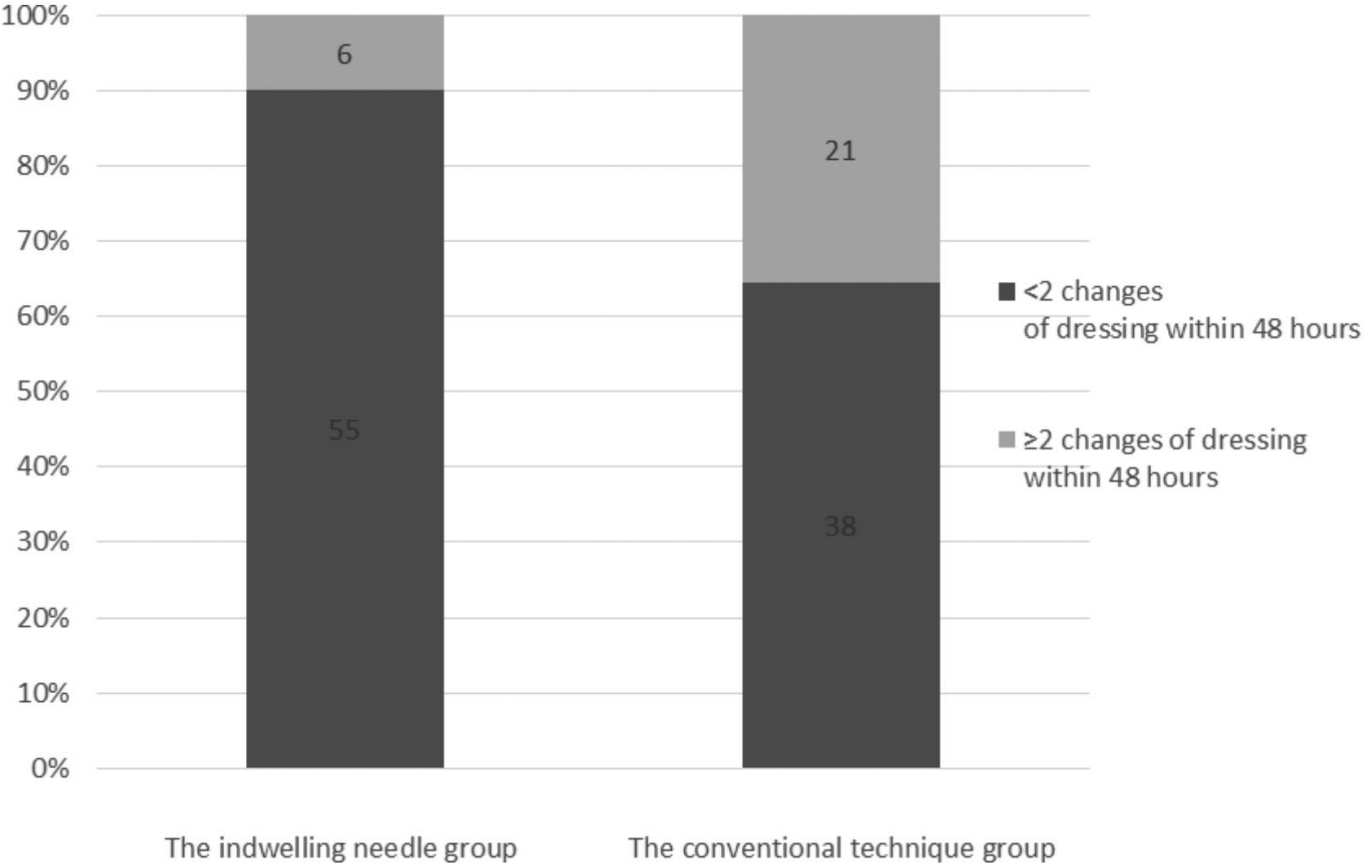
Comparison of dressing changes within 48 h after ECC between the two groups.

The risk of bleeding complications of ECC was compared between the two groups. In the indwelling needle group, there were 55 cases (90.2%) with one change of dressing within 48 h. There were six cases (9.8%) with ≥2 changes of dressing within 48 h. In the conventional technique group, there were 38 cases (63.3%) with one change of dressing within 48 h. There were 21 cases (35.0%) with ≥2 changes of dressing within 48 h, which was significantly more than that in the indwelling needle group (*p* = 0.001). Hence, ECC with indwelling needle can effectively improve the risk of catheter-related bleeding (Figure 2).

Comparison of the final outcomes of CRBSI and CLABSI between the two groups showed no significant differences in CRBSI and CLABSI between the two groups (*p* > 0.05) (Tables 3, 4).

**Table 3:** Comparison of the incidence of CRBSI between the two groups.

Groups	*n*	CRBSIs (*n*)	Catheter indwelling duration (day)	CRBSIs/1,000 catheter days	*p-*value
The indwelling needle group	61	1	1,321	0.76 (1/1,321)	0.619
The conventional technique group	59	2	1,524	1.31 (2/1,524)	

**Table 4:** Comparison of the incidence of CLABSI between the two groups.

Groups	*n*	CLABSIs (*n*)	Catheter indwelling duration (day)	CRBSIs/1,000 catheter days	*p-*value
The indwelling needle group	61	4	1,321	3.03 (4/1,321)	0.058
The conventional technique group	59	11	1,524	7.22 (11/1,524)	

## Discussion

In this study, the puncture times, catheter puncture time, bleeding after catheterization, catheter indwelling time and complications of VLBW were retrospectively analyzed and compared between the indwelling needle group and the conventional technique group of infants admitted at the NICU of the Children's Hospital affiliated to Zhejiang University School of Medicine. The success rate of first attempt cannulation and incidence of bleeding after catheterization in the indwelling needle group was significantly higher than that in the conventional technique group, which provided an ideal solution for the difficulty of ECC in VLBW infants and excessive bleeding after puncture.

The conventional puncture technique of ECC is to insert the catheter through the hollow tunnel of the sheath after successful puncture, and then tear off the sheath. The hollow and tearable design of the sheath solves the problem of access during catheterization and removal of the sheath after catheterization. However, in order to meet the requirement of passing through a 1.9 Fr catheter, the diameter of the sheath is much larger than that of general venipuncture, which increases the difficulty of successful venipuncture. The success of the puncture of the sheath also directly affects the success rate of the catheterization. INS recommends that the smallest diameter catheter should be selected for meeting clinical needs. The outer diameter of the 24G indwelling needle is significantly smaller than that of the 20G sheath, which is more suitable for ECC in VLBW infants (14, 15), increases the success rate of venipuncture and minimizes implant-related injury.

The introduction of the catheter through a 1.9 Fr ECC built-in stylet based on the method reported by Seldinger (16) is the key aspect of this technological innovation. Whether the placement of the stylet into the vessel leads to increased phlebitis due to vascular injury remains unknown. However, the recurrence of phlebitis is related to many factors including the damage to blood vessels caused by punctures, the diameter of the blood vessels, and the number of catheter delivery times (17). Since the first appearance in 1956, the Seldinger's method has been used for more than half a century and there is no report on the increased incidence of phlebitis caused by the stylet in the blood vessel. In this study, none of the 120 successfully catheterized VLBW infants developed phlebitis. Thus, the introduction of the catheter through the built-in stylet did not increase the incidence of phlebitis. However, the puncture of indwelling needles with a diameter more than 20G could cause phlebitis (18). Due to the small sample size of this study, there was no difference between the two groups. Larger sample size studies are needed to confirm that our method is more advantageous in reducing the incidence of phlebitis.

Causes of catheter-related infection are complex. Long puncture time and multiple punctures are risk factors for increased catheter-related infection (19, 20). Faunes Pérez M et al. reported that every additional puncture increases the risk of infection by 2.1 times (21). It is also reported that the incidence of CRBSI or CLABSI in NICUs in developing countries is higher than that in developed countries. The incidence of CLABSI in NICUs in China is (5.20–7.35)/1,000 catheter days (22). The incidence rate of CRBSI in NICU of a tertiary hospital in the United States was (0.6–2.1)/1,000 catheter days, and the incidence rate of CLABSI in NICUs in developed countries in Europe was (1.8–8.4)/1,000 catheter days (23, 24). The incidence of CRBSI at our institution was (0.05–1)/1,000 catheter days, and the incidence of CLABSI was (5–8)/1,000 catheter days. In this study, the incidences of CRBSI and CLABSI between the two groups were within the range of daily monitoring data of this institution with no significant differences, indicating that ECC with indwelling needles did not increase catheter-related infections. The indwelling needle group showed improved success rate of first attempt cannulation of ECC and shortened catheterization time. Hence, a large sample size study is needed to confirm that the new technology of ECC with indwelling needles can reduce the early infection of ECC.

## Strengths and limitations

This study retrospectively analyzed the effect of the ECC method in VLBW infants at our institution, and compared the advantages and disadvantages between the two groups. The technique is described in detail, and innovations are made according to the characteristics of veins and clinical difficulties in VLBW infants, which provides new solutions for solving practical clinical problems. This technique can be used as reference by NICU colleagues worldwide, and has broad application prospects. However, since this was a single-center, retrospective cohort, non-randomized controlled study based on available data with limited sample size, and there may be selection bias. Hence, more studies are needed on the effects of ECC with indwelling needles.

## Conclusion

Application of ECC with 24G indwelling needles in VLBW infants can improve the success rate of first attempt cannulation of ECC and reduce the risk of bleeding, without increasing the incidence of phlebitis and catheter-related bloodstream infections. This method is safe and effective for VLBW infants undergoing ECC. In addition, this method can also be used for ECC in children with difficult intravenous access (DIVA) to reduce the number of punctures, improve the success rate, and protect the exhausted peripheral veins.

## Data Availability

The original contributions presented in the study are included in the article, further inquiries can be directed to the corresponding author/s.
